# Helium nanodroplets as an efficient tool to investigate hydrogen attachment to alkali cations†

**DOI:** 10.1039/d2cp03841b

**Published:** 2022-11-17

**Authors:** Siegfried Kollotzek, José Campos-Martínez, Massimiliano Bartolomei, Fernando Pirani, Lukas Tiefenthaler, Marta I. Hernández, Teresa Lázaro, Eva Zunzunegui-Bru, Tomás González-Lezana, José Bretón, Javier Hernández-Rojas, Olof Echt, Paul Scheier

**Affiliations:** University of Innsbruck, Institute for Ion Physics and Applied Physics Innsbruck Austria siegfried.kollotzek@uibk.ac.at; Instituto de Física Fundamental, C.S.I.C. Madrid Spain jcm@iff.csic.es; Dipartimento di Chimica, Biologia e Biotecnologie, Università di Perugia Perugia Italy; Departamento de Física and IUdEA, Universidad de La Laguna La Laguna Tenerife Spain; Department of Physics, University of New Hampshire Durham NH 03824 USA

## Abstract

We report a novel method to reversibly attach and detach hydrogen molecules to positively charged sodium clusters formed inside a helium nanodroplet host matrix. It is based on the controlled production of multiply charged helium droplets which, after picking up sodium atoms and exposure to H_2_ vapor, lead to the formation of Na_*m*_^+^(H_2_)_*n*_ clusters, whose population was accurately measured using a time-of-flight mass spectrometer. The mass spectra reveal particularly favorable Na^+^(H_2_)_*n*_ and Na_2_^+^(H_2_)_*n*_ clusters for specific “magic” numbers of attached hydrogen molecules. The energies and structures of these clusters have been investigated by means of quantum-mechanical calculations employing analytical interaction potentials based on *ab initio* electronic structure calculations. A good agreement is found between the experimental and the theoretical magic numbers.

## Abstract 2

Combined experimental and theoretical investigation of molecular hydrogen attachment to sodium cations at sub-kelvin temperatures.

## Graphical abstract

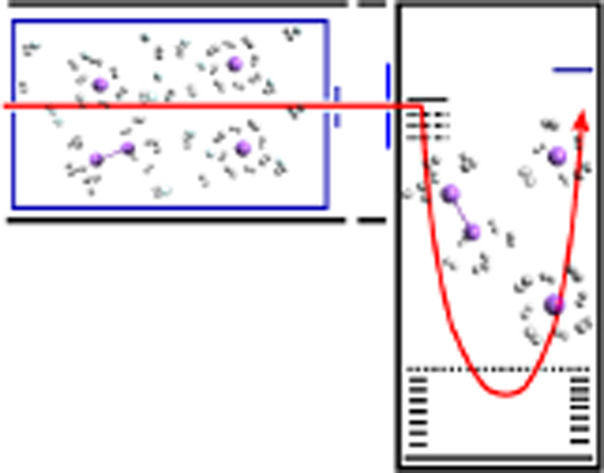

## 1. Introduction

In the ample search for suitable materials for hydrogen storage leading to more sustainable and green energy and fuels, the important role that nanoporous materials might play in the near future^1,2^ and the relevance of metal decoration in improving the performance of such materials have been recognized.^3–5^ Studies have proved that the adsorption of molecular hydrogen gas on graphite, graphene, carbon nanotubes, layers of fullerenes, and other polycyclic aromatic hydrocarbon (PAH) materials is significantly improved by doping the complex with alkali atoms.^6^ Sodium is already well known for being a promising candidate for increasing the efficiency of hydrogen attachment in multiple storage systems and a good alternative to more scarce elements like lithium.^7,8^

Therefore the investigation and characterization of the hydrogen–sodium interaction are important to the H_2_ energy community, driven by the need to develop fundamentally new ways to store hydrogen in low-weight environments with high storage density.^9^ The physisorption energy of H_2_ in pristine carbon-based materials is only around 40–50 meV and ∼30 meV in coronene,^10^ so the goal of doped complexes would be to raise the adsorption energies sufficiently to allow for efficient storage at moderate pressures near ambient temperatures.^11,12^ In line with this, the characterization of the isolated cation sites and their ability to attach H_2_ molecules^13^ are very important since it allows us to very accurately determine the structures and interactions intervening in the hydrogen storage on nanoporous materials.

Along with a high number of already established methods of investigating this process,^14^ this work opens up a new approach using a well-tested technique in laboratory astrochemical investigations.^15–18^ Superfluid helium droplets provide a powerful and flexible environment for investigations of hydrogen interactions with single molecules and dopants. With this different approach, complete knowledge of the most stable structures and configurations can be obtained. Laimer *et al.* reported in 2019 on the production and stability of highly charged droplets of superfluid helium.^19^ The potential of these multiply charged helium droplets led to the construction of a new experiment, which enables a more intense and controlled investigation of processes including the nucleation of dopant cluster ions as well as their decoration with helium or molecular hydrogen^20^ inside of these superfluid helium nanodroplets at a temperature of 0.37 K.^21^ This experimental setup allows accurate and reproducible control of the number of the attached hydrogen molecules. The resulting charged sodium/hydrogen complexes are analyzed using time-of-flight mass spectrometry and shell closures for the attachment of hydrogen are determined *via* local maxima in the ion yield of these species.

In this work, we present experimental as well as theoretical data showing the relative ion abundance of up to 15 hydrogen molecules attached to either a monomer or sodium dimer cations. They are investigated in consideration of their ability to reversibly bind molecular hydrogen. The relative ion abundances of Na^+^(H_2_)_*n*_ and Na_2_^+^(H_2_)_*n*_ from mass spectra obtained by sequential pickup of sodium and molecular hydrogen into multiply charged helium nanodroplets are compared with quantum mechanical calculations. The computational results provide detailed information about the binding energy and structure of a specific number of H_2_ molecules attached to a positively charged sodium atom or dimer. The structure of the paper is as follows: Section 2 outlines the methods employed, both experimental and theoretical; in Section 3 we analyze the experimental data and present the theoretical results; Section 4 is devoted to conclusions.

## 2. Methods

### 2.1. Experimental

**Fig. 1:**
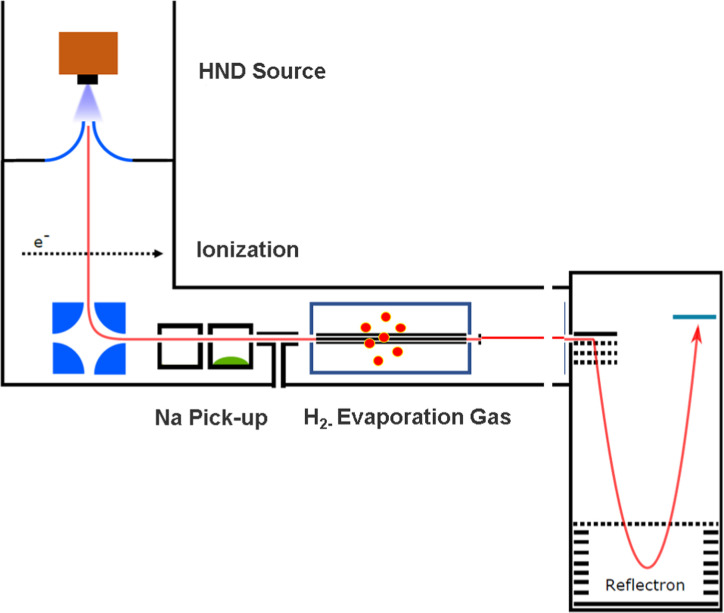
The experimental setup used to decorate Na^+^ and Na_2_^+^ sodium cations with H_2_ molecules embedded in helium nanodroplets (HNDs). The ionized and size-to-charge selected HNDs are first doped with sodium in the pick up chamber. Subsequent pick up of molecular hydrogen leads to a replacement of helium with H_2_ and to the solvation of the sodium ions with hydrogen. Furthermore, the helium matrix is then stripped from the embedded cluster ions by collisions with H_2_ gas at ambient temperature in the evaporation cell.

The experimental setup utilized in this work has been explained in detail elsewhere,^22^ but a brief description is given below. Helium nanodroplets (HNDs) are produced *via* supersonic expansion of ultrapure helium (Messer, purity 99.9999%) with a stagnation pressure of 20 bar through a 5 μm pinhole nozzle into ultrahigh vacuum. The nozzle was cooled with a closed-cycle cryocooler (Sumitomo Heavy industries) and counter-heated with an ohmic resistor operated with a PID controller (Lakeshore Model331) to 8.8 K. Under the present conditions the pressure in the source chamber increases from 0.01 mPa to 53 mPa. According to Gomez *et al.*^23^ and Laimer *et al.*^19^ the resulting average droplet size is calculated to be 5 × 10^6^ He atoms under the present conditions. To prevent the destruction of HNDs by collisions with shock fronts, the resulting jet of He was then passed through a 0.5 mm skimmer (Beam Dynamics, Inc) located 10 mm after the nozzle. HNDs are ionized by a Nier-type electron impact ionization unit directly after the skimmer. The ion source was operated at an electron energy of 65 eV and an electron current of 350 mA. Under these conditions most HNDs are hit by multiple electrons that can directly ionize helium atoms; the positively charged He^+^ moves to the center of the droplet and, after several steps, ends forming He_2_^+^. This ion is subsequently solvated by neighboring He atoms leading to tight He_3_^+^ structures.^24^ The tree atoms ionic core then forms the so-called Atkins snowball.^25,26^ Mutual repulsion of these He_*n*_^+^ charge centers into minimum energy configurations distributes them uniformly close to the surface of HNDs.^27^ This results in a mean charge state of the selected HNDs of *z* = 15. Charged droplets, consisting of 3 × 10^5^ He atoms per charge were then mass-per-charge filtered by an electrostatic quadrupole bender. Subsequent guiding of the charged HNDs through sodium vapor leads to the pick-up of single atoms. Due to the heliophobic nature of sodium atoms, they stay unsubmerged on the surface of the HNDs until a certain limit is reached.^28^ The polarizability of captured sodium is much higher than that of He and therefore, ion-induced dipole interaction attracts dopants to the charge centers also sitting close to the surface of the droplet. Charge transfer from He_*n*_^+^ to the first dopant leads to a Na^+^ charge center and the excess energy due to the difference in the ionization energies will be dissipated into the HNDs and leads to the evaporation of 1600 He atoms per eV. Further dopants will simply attach to an already existing ionic core and the binding energy will be transferred to the surrounding He, leading to the evaporation of additional He atoms from the surface of the HND. Subsequent collisions with room temperature H_2_ vapor first replaces He atoms surrounding the sodium ions with hydrogen molecules and upon consecutive collisions with H_2_ the droplet shrinks below the critical size for the given charge state and a hydrogen solvated sodium cation is ejected from the droplet. In the next section, we will compare binding energies for the cluster interactions in the cases of He and H_2_, and the easy replacement of the first by the second species will then be easily understood. With increasing hydrogen pressure in this evaporation cell (see Fig. 1) the amount of H_2_ molecules solvating the sodium ions can be reduced appropriately to enhance the visibilty of intensity anomalies and shell closures. This effect can be readily noticed in the ESI,† Fig. S1, where several pressures of hydrogen can be seen and its influence in the population of different sodium clusters. The hydrogen solvated sodium ions are analyzed using a time-of-flight mass spectrometer (Q-TOF Ultima Waters/Micromass).

**Fig. 2:**
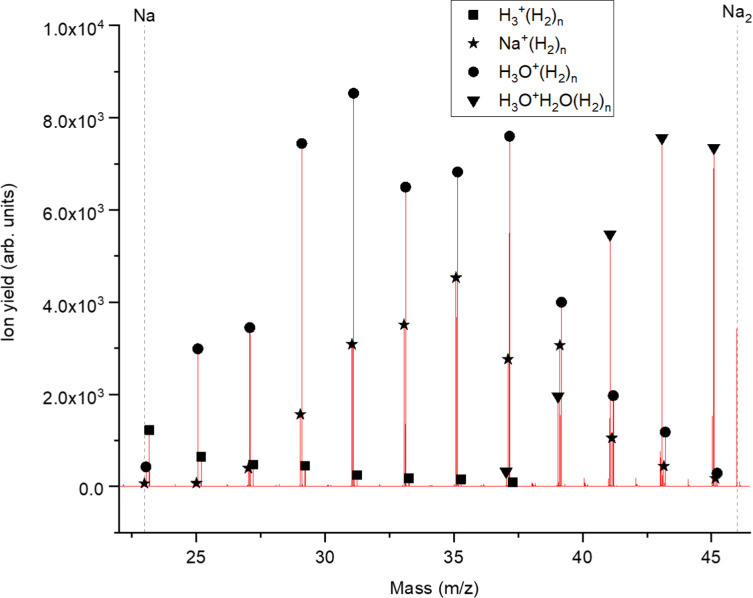
Example of a mass spectrum showing the H_2_ decoration of positively charged sodium monomers. A hydrogen pressure of 40 mPa leads to an environment where the pure hydrogen series as well as the hydrogen decoration of water clusters is visible. The exact mass of the impurities never overlaps with the slightly less heavy sodium series and is therefore not influencing its intensity distribution. On the left side of the spectrum the pure hydrogen series is still present (squares) and on the right half the water dimer (triangle) series is starting. The hydrogen decoration of the sodium cation is labeled with stars.

Fig. 2 displays a section of mass spectrum obtained by the method described above. A larger range of masses (*m*/*z*) that can be reached in the experiment can be found in the ESI,† Fig. S2. Important mass peaks are the bare sodium monomer (*m*/*z* = 23) and dimer (*m*/*z* = 46) cations, followed by mass peaks due to Na_1,2_^+^(H_2_)_*n*_. In the mass spectrum sodium cluster ions Na_*m*_^+^ up to sizes of *n* = 13 can be identified and the corresponding mass peaks are indicated in Fig. 2 by the dashed vertical lines (as well as in Fig. S2 in the ESI†). Under the present conditions, where sodium cluster ions are solvated by hydrogen molecules, the cluster size distribution of Na_*m*_^+^ does not exhibit the well-known intensity anomalies often reported for cationic alkali clusters,^29,30^ however, several Na_*m*_^+^(H_2_)_*n*_ cluster size distributions exhibit clear intensity anomalies as a function of the number of hydrogen molecules *n*. The mass spectra for different pressures in the evaporation cell were measured to confirm the magic numbers of the Na^+^(H_2_) and Na_2_^+^(H_2_)_*n*_ cluster size distributions shown in the results.

The setup is easily adjustable and in order to rule out pick up effects, experiments with H_2_ predoping and helium as an evaporation gas where performed. This alternative sequence leads to the same cluster products and similar relative ion abundances as the previous pick up sequence. To probe the possible underlying mechanism in the alternative pick up sequence, we carried out high level *ab initio* calculations. When sodium is picked up first, as in the regular sequence, the main pathway would follow a charge transfer from the HND to sodium (Na^+^) with subsequent addition of molecular hydrogen, resulting in the mass spectrum shown in Fig. 2. On the other hand, with a reversed pickup sequence in which H_2_ doping is achieved first, charge transfer leads to H_2_^+^ and *via* pickup of additional hydrogen molecules to H_3_^+^ as the first charge carrier (see ref. 20 for an explanation leading to this triatomic species), this species can further encounter Na with two possible outcomes,

1 H_3_^+^ + Na → NaH + H_2_^+^ Δ(*E*) = 4.628 eV

2 H_3_^+^ + Na → Na^+^ + H_2_ + H Δ(*E*) = −4.053 eV

It can be seen that the theoretical electronic energy difference (obtained from reactants and products optimizations at the MP2/aug-cc-pVTZ level of theory) clearly favors the second pathway, thus suggesting an explanation of why both pick up sequences lead to the same results. Since NaH^+^ is not a chemical species but rather an aggregate and H in eqn (2) is more weakly attached to the Na^+^ cation than the corresponding diatomic, H_2_, it is preferentially removed from the cluster upon collision induced activation. This explains the low abundance of the Na_1,2_^+^(H_2_)_*n*_H peak series. It is also worth noting that for similar clusters of Cs^+^(H_2_)_*n*_ formed in HNDs, no CsH^+^ species were detected.^31^ A mass spectrum corroborating this fact can be found in the ESI,† Fig. S3.

### 2.2. Theoretical methods

#### 2.2.1. Potential interaction

In order to provide an adequate theoretical description of the experimental findings, we first need to produce a suitable interaction potential for clusters containing several hydrogen molecules and cationic sodium (Na^+^ or Na_2_^+^) species.

We have taken for the total interaction potential a sum of two-body (2B) terms and thus we write for (H_2_)_*n*_Na_*m*_^+^3
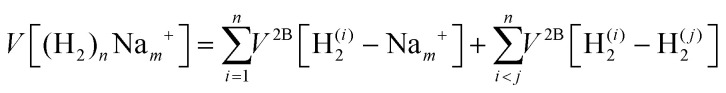
where *n* runs from [1–14] and *m* = 1,2, for Na^+^ or Na_2_^+^. In the case of the monocation, Na^+^, we have also studied the influence of three-body (3B) interactions, by adding an extra term 
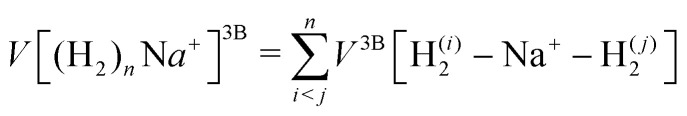
.

The different contributions are represented by suitable functional forms,^31,32^ whose parameters are optimized on accurate quantum *ab initio* estimations^33–35^ for both interaction energies and monomer properties. A full account of the procedure and parameters to describe the complete force field is given in the ESI.†

An important point to be emphasized is the different representation of the long range in Na^+^(H_2_)_*n*_ and Na_2_^+^(H_2_)_*n*_ clusters stability which is controlled by the induction attraction. In particular, while Na^+^ is a charge point,^36–38^ Na_2_^+^ is a strongly polarizable and elongated ionic diatom^39,40^ showing an equilibrium distance, *R*_e_, of 3.71 Å and a strongly anisotropic charge distribution, as suggested by its high electric quadrupole moment (see the ESI†). Therefore, the radial dependence of the induction attraction for configurations involving Na_2_^+^ aligned along the intermolecular distance (see configurations *L* and *T*_b_ in the Potential Energy Surfaces section of the ESI†) assumes the canonical *R*^−4^ dependence (such as that for Na^+^) only asymptotically (*R*⪢*R*_e_), while in the region of interest for cluster stability (*R* ∼ *R*_e_) the *R*^−6^ dependence, typical of a dipole, is found to be more appropriate (see Fig. S4 in the ESI†).

The expressions of the different terms in eqn (3) as well as a full account of the procedure and parameters to describe the complete force field is given in the ESI.†

#### 2.2.2. Cluster energies and structures

Once a reliable potential interaction function has been formulated, our goal is to determine the structure and stability of the different clusters for increasing values of H_2_ monomers. The enhanced ion yield of the current experimental set-up together with the extraordinary accuracy reached in the mass spectra prompt us to take advantage of these data sets and to try the best theoretical framework to study these clusters. To this end we will make use of Path Integral Monte Carlo calculations (PIMC) in a pseudo-atom model for the H_2_ molecule and a Diffusion Monte Carlo (DMC) approach within a rigid rotor approximation for the mentioned hydrogen monomers. We will also check the importance of the 2B *vs.* 3B terms included in the interaction potential.

In the PIMC, hydrogen molecules will be treated as pseudoatoms.^41,42^ In this method^43^ the analogy in the partition function for a system composed of *N* classical ring polymers, each having *M* beads with the corresponding quantum system of the *N* particles is exploited to obtain the energy and structures of the clusters. We have used the thermodynamic estimator,^44^ and classical minimization procedures such as Evolutionary Algorithm and a Basin-Hoping technique^45,46^ to start sampling the initial configuration. Details of the method and parameters of interest are given in the ESI.†

The DMC method^47,48^ computes the ground state of the cluster by means of a transformation of the time-dependent Schrödinger equation into a diffusion equation by changing the variable time, *t*, to imaginary time, *τ* = it. The ground state of the system is then achieved as a lasting term in the (imaginary time) propagation of the diffusion equation. In this method, the wavefunction is represented by a set of replicas describing different configurations of the particles of the system and, at each time step Δ*τ*, the particles randomly move according to the kinetic energy term in the Hamiltonian and the replicas multiply or disappear with a probability depending on the value of the potential energy term.^47,48^ We have used the implementation for rigid bodies due to Buch and collaborators^49–51^ and a classical Monte Carlo (MC) method to obtain the initial configurations for the DMC calculations. The working parameters and details on the calculations can be found in the ESI.† We have used for all the calculations 1.007825 amu and 22.98977 amu for the masses of H and Na atoms, respectively.

**Fig. 3:**
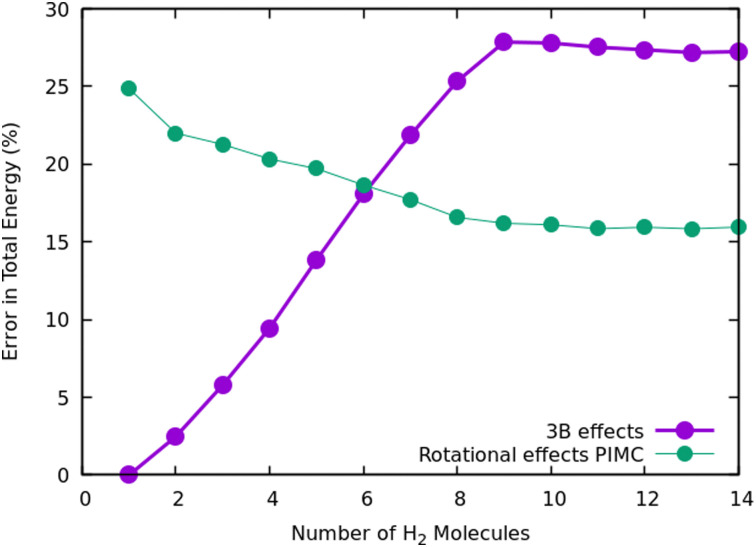
Error in the estimation of cluster energies when a pseudoatom approximation is used, or when only 2B terms are employed for the interaction potential. In both cases it is assumed that the “exact” result is the one computed within the rigid rotor approximation and the additional use of a 3B term.

**Fig. 4:**
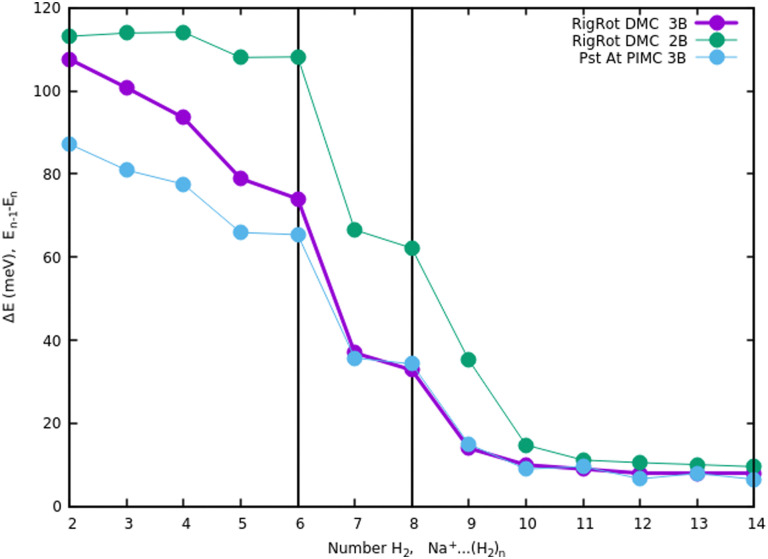
Evaporation energies, for Na^+^(H_2_)_*n*_, within several approaches, DMC with a rigid rotor model, 2B and 3B interaction potential and PIMC with a 3B potential.

Once we have briefly described the theoretical methods, we present in Fig. 3 the percentage of differences found in the cluster total energy when we use a pseudo-atom approach or we just take the 2B terms in the potential interaction for the case of Na^+^(H_2_)_*n*_. A comparison is made with the rigid-rotor DMC approach including the 3B terms considered as the “exact” result. We can see that the error in total energy stabilizes very quickly and for *n* ≥ 8 it becomes practically constant. These differences correspond to the total energy but, actually, when we consider energy differences known as evaporation energies, shown in Fig. 4, it can be appreciated that the main features regarding peaks, plateaus and slopes remain unchanged, and would lead to similar structures or magic numbers. In particular it can be observed that the 3B effects are more noticeable and that a pseudoatom approach, except for the smaller hydrogen clusters, brings no significant differences. This could be anticipated from Fig. 3 where the differences remain almost constant with a very small slope, for the pseudoatom approach.

Therefore our rather thorough analysis indicates that for larger clusters a simpler treatment (2B interactions only, or considering a system composed of pseudo atoms) can still yield accurate predictions, regarding structure and stabilities. For analysis of the results and comparison with the experiment in the next section, we will use DMC within the rigid rotor model and the 3B interaction potential for a sodium monomer, while a 2B interaction model will be used for the sodium dimer clusters.

At this point it is also useful to stress that dissociation energies computed in this work *E*_0_ = −112.28 meV for Na^+^H_2_ and *E*_0_ = −38.86 meV for Na_2_^+^H_2_ dimers, are larger than those previously found for Na^+^He (*E*_0_ = 32.61 meV in the harmonic approximation in ref. 52) and Na_2_^+^He (*E*_0_ = −6.1 meV,^53,54^*E*_0_ = −7.0 meV^55^), of about a factor of 3 and 6, respectively. These differences result in the easy replacement of He by H_2_ in the evaporation cell and are mostly due to the higher polarizability of H_2_ compared with He, even if the electrostatic contribution which is absent for adducts involving He plays a non-negligible role.

## 3. Data analysis and results

**Fig. 5:**
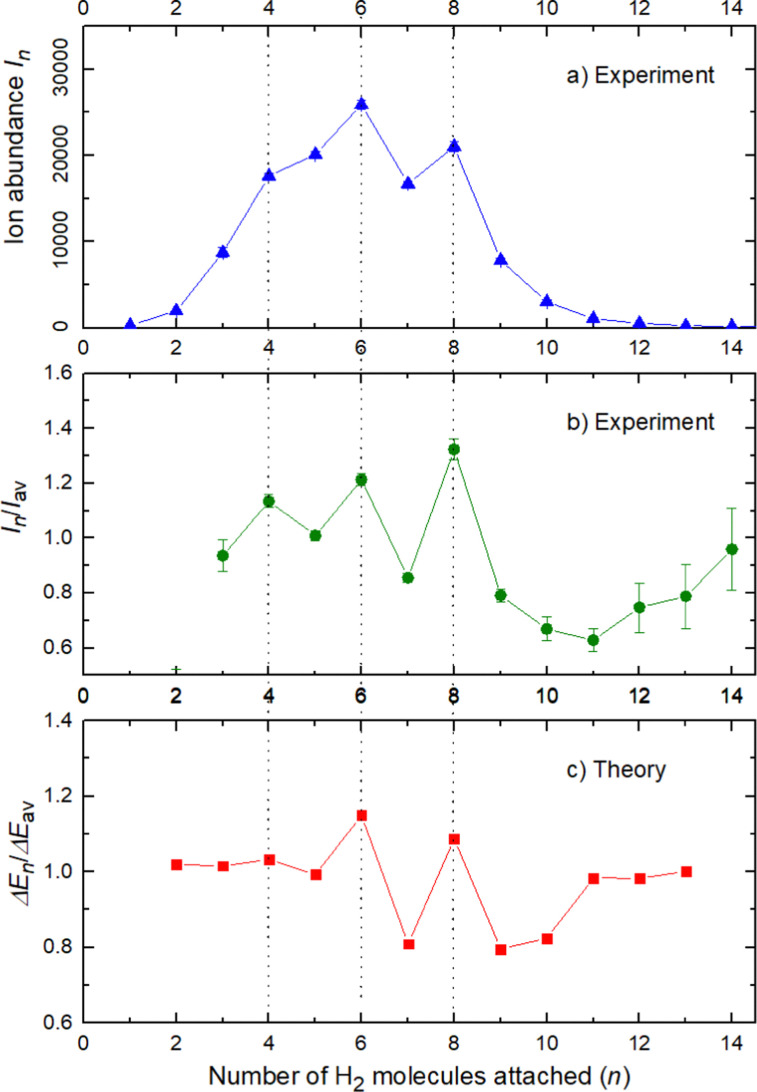
Data for Na^+^(H_2_)_*n*_. Panel a: experimental ion abundance *versus* size, *n*. Panel b: experimental ion abundance *I*_*n*_ divided by its local average, *I*_av_. Panel c: evaporation energy Δ*E*_*n*_ calculated with the rigid rotor model DMC, divided by its local average Δ*E*_av_. Note the similarity of the data in panels b and c.

**Fig. 6:**
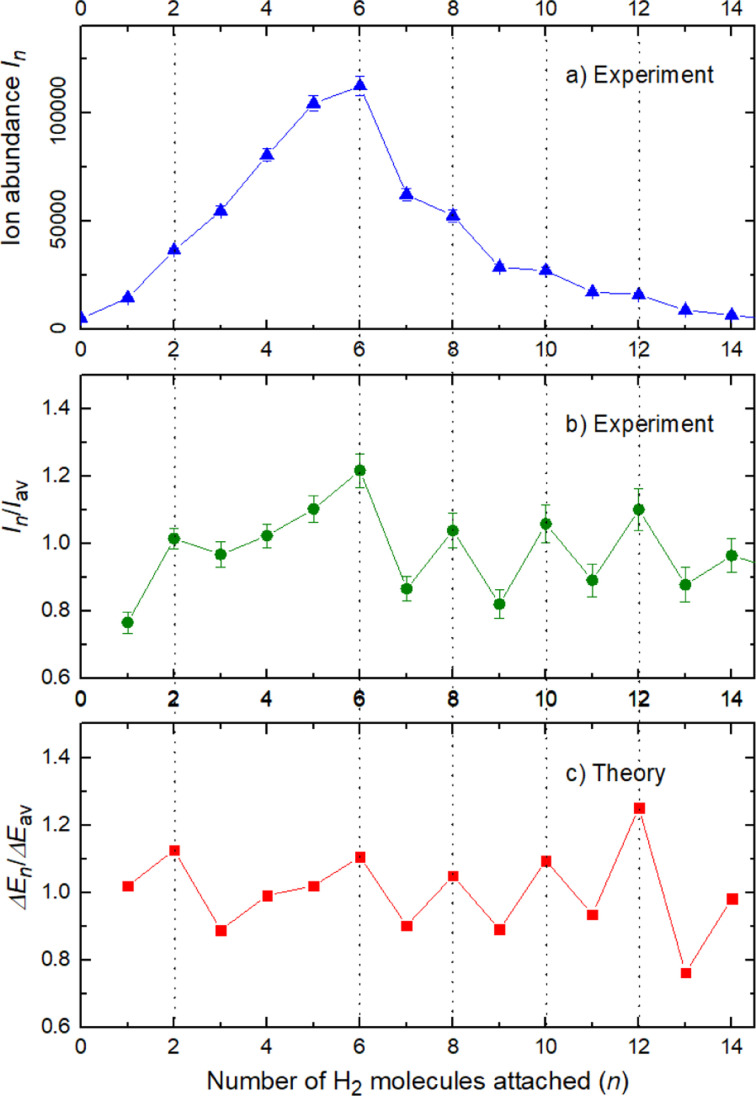
Na_2_^+^(H_2_)_*n*_. Panel a: experimental ion abundance *versus* size, *n*. Panel b: experimental ion abundance *I*_*n*_ divided by its local average, *I*_av_. Panel c: evaporation energy Δ*E*_*n*_ calculated with the rigid rotor model DMC, divided by its local average Δ*E*_av_. Note the similarity of the data in panels b and c.

The experimental ion abundance *I*_*n*_ is plotted *versus* the number of the attached H_2_ molecules, *n*, in Fig. 5a and Fig. 6a for Na^+^(H_2_)_*n*_ and Na_2_^+^(H_2_)_*n*_, respectively. The data shown in these figures are extracted from the mass spectra shown in Fig. 2. The ion abundances feature local anomalies which suggest similar local effects in the evaporation energies Δ*E*_*n*_, for example at *n* = 6 and 8 for Na^+^(H_2_)_*n*_. Several procedures have been proposed in the literature in order to establish a quantitative relationship between anomalies in *I*_*n*_ and Δ*E*_*n*_; their applicability depends on the experimental conditions. For the present data we adopt an approach that was first proposed by Leidlmair *et al.*,^56^ and justified in more detail in ref. 57 and 58. It asserts that4
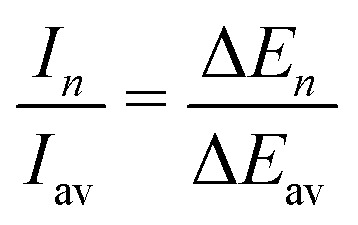
where *I*_av_ and Δ*E*_av_ are local averages of *I*_*n*_ and Δ*E*_*n*_, respectively. Eqn (4) is supposed to be valid, approximately, if two conditions are met: (i) The observed cluster ions are the unimolecular fragments of larger precursors, and (ii) the heat capacity of the observed cluster ions is close to zero, *i.e.* their vibrational modes are not excited at the relevant temperature. Condition (i) is clearly met because the hydrogen gas in the collision cell is at room temperature; the cluster ions that are extracted from the collision cell have ample time to cool by evaporation, reaching a temperature *T* ≈ Δ*E*_*n*_/(*G k*_B_) where *G* ≈ 25 is the Gspann parameter.^59,60^ The most tightly bound cluster ions considered here have Δ*E*_*n*_ ≈ 0.1 eV which corresponds to *T* ≈ 50 K. Condition (ii) is also likely to be met: Intramolecular vibrations of H_2_ are certainly not excited at or below 50 K, and the small mass of H_2_ results in large intermolecular vibrational frequencies in the sodium–hydrogen complexes.

The left and right-hand sides of eqn (4) are plotted in Fig. 5b and c, respectively, for Na^+^(H_2_)_*n*_. The local averages *I*_av_ and Δ*E*_av_ were obtained by averaging over adjacent sizes with weights computed from a Gaussian with a standard deviation *σ* = 1 (the results did not change significantly if *σ* = 2 was chosen). The similarity of the data in Fig. 5b and c is excellent. Cluster ions with *n* = 6 and 8 clearly form magic numbers, although the local maximum at *n* = 4 in the experimental data is not completely supported by the theory. Likewise, experimental and theoretical data for Na_2_^+^(H_2_)_*n*_ are plotted in Fig. 6b and c, respectively. Again, the two data sets are very similar; they feature local maxima at *n* = 2, 6, 8, 10, and 12.

These evaluations are also performed for different H_2_ pressures in order to exclude anomalies due to size distribution effects and the results for nearly all pressures are similar to the ones previously shown and in fact the survival of the higher ion yield at several pressures is also an indication of larger stability, or in other words, magic numbers.

The appearance of exceptionally stable structures or “magic numbers” with agreement between theory and experiment deserves some comments, specially concerning the differences and similarities between the sodium monomer and dimer and their interactions with the H_2_ molecules.

**Fig. 7:**
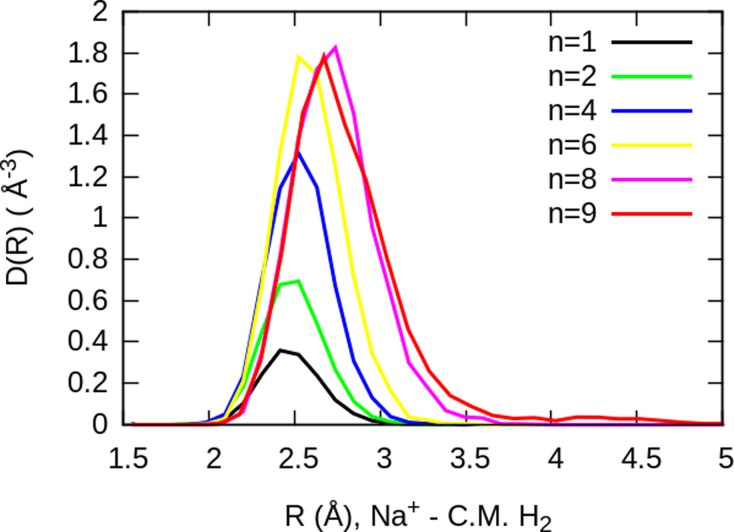
Na^+^(H_2_)_*n*_ radial distributions, normalized to the number of H_2_ molecules, for several cluster sizes. The closing shell at *n* = 8, can be noticed by the small bump that can be appreciated for *n* = 9.

The simplest case corresponds to the sodium monomer, in which three clear islands of stability are apparent, for hydrogen sizes, *n* = 4, 6, and 8, as shown in the bottom of Fig. 5. In Fig. 7 we show the theoretical radial distribution, *D*(*R*), for several compositions of these clusters. The distributions are normalized to the number of H_2_ contained within. It can be seen that up to *n* = 8 the distributions vanish between 3–3.5 Å while for *n* = 9, there is a small recurrence between 4–4.6 Å that corresponds to the beginning of a new shell around the Na^+^ cation. These results are very similar to those found for Li^+^He_*n*_,^41^ where also a close resemblance can be found in the second energy differences for that system, and the one we show at the bottom of Fig. 5. This result is a consequence of the interactions brought into play in both cases, with similar equilibrium distances and well depths between the cation and the other component of the cluster (He/H_2_) that, in turn, have a small interaction between them.

In the structure two angles are relevant, that we will call (*θ*, *Φ*). The angles *θ* in a given cluster are those formed between the vector defining the bond in a particular H_2_ molecule, and the vector from its center of mass to the cation. More interesting for the geometry are the *Φ* angles, which are those formed between two vectors that are directed from the sodium atom, to the center of mass of two different H_2_ units. These angles can be better seen in the ESI,† where we have plotted the minimum energy structures obtained with classical MC simulations, in the case of Na^+^(H_2_)_6_ and Na_2_^+^(H_2_)_12_ for the sodium monomer and dimer, respectively. They have also been included as insets, in some of the next figures.

**Fig. 8:**
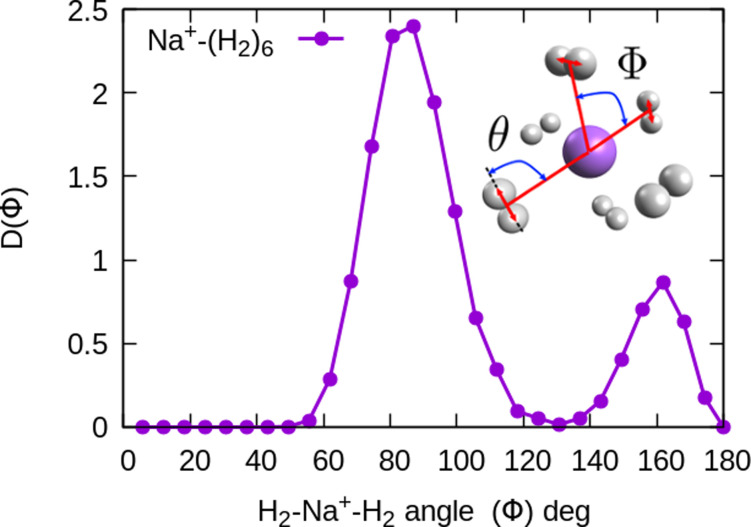
Na^+^(H_2_)_6_ angular distributions of 
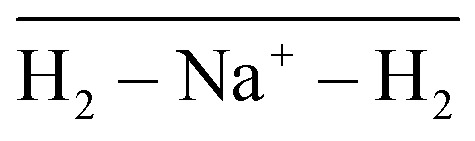
 The inset corresponds to the classical MC structure.

In the case of the sodium monomer, the structure is nearly rigid, the H_2_ molecules orient with their bond perpendicularly to the vector joining each molecular center of mass to the cation, angle *θ* = 90°, with a narrow distribution around this value. On the other hand, how the different monomers are arranged surrounding the cation, is better defined by the angle *Φ* and the distribution for the cluster Na^+^(H_2_)_6_ is shown in Fig. 8. As can be seen, this distribution, *D*(*Φ*), shows two predominant peaks with maxima at angles around 90 and 160 degrees and with little dispersion, certainly signaling a clear structure corresponding to an octahedron, that was also found previously in other clusters.^41,61^ This structure is close to the classical one, which is included as an inset in the figure. Similar tight structures can be found for *n* = 4 and 8, with geometries corresponding to a tetrahedron and square antiprism, respectively. Barbati *et al.*^62^ reported structures obtained from *ab initio* calculations up to *n* = 7 and the same structures were found for *n* = 2, 3, 4, and 6.

**Fig. 9:**
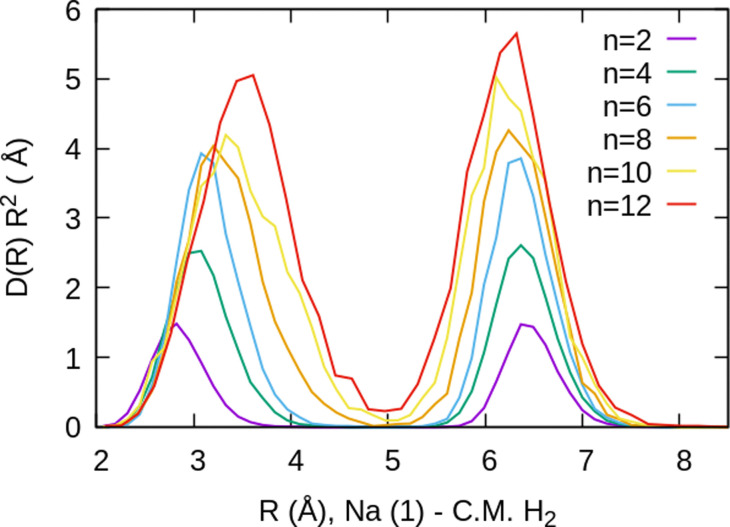
Na_2_^+^(H_2_)_*n*_ radial distributions, normalized to the number of H_2_ molecules, for several cluster sizes, referred to the position of one of the sodium atoms Na(1) which is placed at the origin of the coordinates. The distributions are multiplied by the volume element *R*^2^ to show more clearly that there are similar numbers of monomers in the first and second peaks (around each atom of Na_2_^+^). Note that the second sodium atom, Na(2), is located at a distance of 3.71 Å.

For the case of the sodium dimer clusters, Na_2_^+^(H_2_)_*n*_, we present in Fig. 9 the radial distributions of H_2_ molecules with respect to one of the sodium atoms composing the dimer (Na(1) in Fig. S3 in the ESI†). It can be seen that there are some H_2_ monomers around Na(1), leading to one peak, and at longer distances, the rest of the monomers are more directly joined to the other atom, Na(2), leading to the second peak. The plot indicates that there is a preference for H_2_ to evenly cover each Na atom. This feature has been checked by integration of the distributions of Fig. 9 up to the middle of the two peaks (around 5 Å), and it was found that the number of diatomic molecules around each Na is, in general terms, half the total number of them. This is the origin of the relatively large stability of the clusters with an even number of H_2_ molecules observed both in the calculations as well as in the measurements of Fig. 6.

**Fig. 10:**
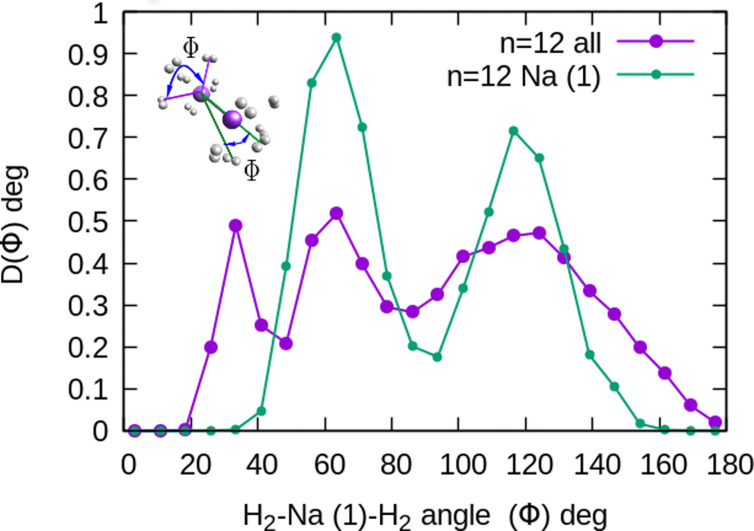
Na_2_^+^(H_2_)_12_ angular distributions of 
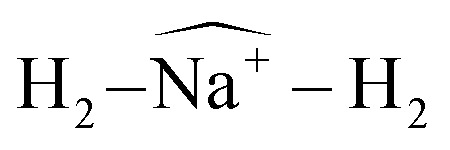
 angles, corresponding to one of the Na atoms in the dimer. The inset corresponds to the classical MC structure, indicating two possible *Φ*′*s* angles. Na(1) is the sodium atom from which the angles are measured.

The existence of a bond in Na_2_^+^, leading to a more anisotropic and weaker interaction with H_2_ as compared with Na^+^, also makes the distribution more prone to forming caps around each alkaline atom, rather than a spherical distribution around a single sodium cation. This effect can be seen in Fig. 10 for *n* = 12, in which we plot (similarly as in Fig. 8) the distribution of angles *Φ* formed between two vectors connecting one of the sodium atoms, Na(1), and the center of mass of any two hydrogen molecules. The peak around *Φ* = 30° corresponds to H_2_ diatoms which are around the other Na atom, Na(2). If we discard the H_2_ monomers surrounding that atom (equivalent to eliminating the peak on the right of Fig. 9), we get a bimodal distribution (green curve in Fig. 10 whose central angles correspond to six molecules forming the cap of an icosahedron (for comparison, see ref. 31 for the icosahedral structure found for Cs^+^(H_2_)_12_). This structure is close to the classical one, shown as an inset in Fig. 10.

## 4. Conclusions

In conclusion, this work shows an important mechanism for the future understanding of how molecular hydrogen binds to single sodium atoms. The good agreement between the experimental and theoretical data supports the idea of favored structures, where the attachment of 4, 6 and 8 hydrogen molecules is especially strong for the sodium monomer Fig. 5. The dimer complexes show agreement between theory and experiment regarding the special stability of complexes with 2, 6, 8, 10 and 12 molecules, since both Na atoms behave as quasi-independent attractors for H_2_ molecules, each of them showing the same number of stable molecule attachments. This symmetric hydrogen attachment to each side of the sodium dimer leads to two-step-plateaus (5,6–7,8–9,10–11,12) for ion yields, shown at the top of Fig. 6, that can also be observed in evaporation energies. Our calculations, concerning the energy difference of every possible pathway for the generation of the cluster complexes due to the experimental setup and its pick up sequence, conclusively explain the ion abundances observed. These calculations also indicate that protonation of single sodium atoms is very unlikely and that the binding energy of a hydrogen molecule is higher than the one of a single H atom, what explains the low abundance of sodium cluster ion complexes with an odd number of hydrogen atoms.

The theoretical calculations confirm and support these conclusions and explain the behavior of the monomer *versus* the sodium dimer. The present results are expected to be of relevance for organic materials containing alkali atoms, where electron transfer to the organic component leads to positively charged alkali ions that act as attractors for hydrogen molecules in novel hydrogen storage materials.^6^

## Data availability

The data that support the findings of this study are available from the corresponding authors upon reasonable request.

## Author contributions

S. Kollotzek, S. Tiefenthaler, and P. Scheier carried out the experiments. J. Campos-Martnez, M. Bartolomei, F. Pirani, M. I. Hernández, T. Lázaro, E. Zunzunegui-Bru, T. González-Lezana, J. Bretón, and J. Hernández-Rojas worked on the theoretical results. O. Echt participated in the experiments and the comparison with the theoretical results.

## Conflicts of interest

We declare no conflicts of interest.

## Supplementary Material

CP-025-D2CP03841B-s001
