# Correction: Helium nanodroplets as an efficient tool to investigate hydrogen attachment to alkali cations

**DOI:** 10.1039/d3cp90119j

**Published:** 2023-06-06

**Authors:** Siegfried Kollotzek, José Campos-Martínez, Massimiliano Bartolomei, Fernando Pirani, Lukas Tiefenthaler, Marta I. Hernández, Teresa Lázaro, Eva Zunzunegui-Bru, Tomás González-Lezana, José Bretón, Javier Hernández-Rojas, Olof Echt, Paul Scheier

**Affiliations:** a University of Innsbruck, Institute for Ion Physics and Applied Physics Innsbruck Austria siegfried.kollotzek@uibk.ac.at; b Instituto de Física Fundamental, C.S.I.C. Madrid Spain jcm@iff.csic.es; c Dipartimento di Chimica, Biologia e Biotecnologie, Università di Perugia Perugia Italy; d Departamento de Física and IUdEA, Universidad de La Laguna La Laguna Tenerife Spain; e Department of Physics, University of New Hampshire Durham NH 03824 USA

## Abstract

Correction for ‘Helium nanodroplets as an efficient tool to investigate hydrogen attachment to alkali cations’ by Siegfried Kollotzek *et al.*, *Phys. Chem. Chem. Phys.*, 2023, **25**, 462–470, https://doi.org/10.1039/D2CP03841B.

In the published version of this manuscript, acknowledgement for one of the funding sources was inadvertently omitted. The authors would like to include “S. K. is supported by the Austrian Science Fund (FWF) W1259-N27” in the Acknowledgements.

The Royal Society of Chemistry apologises for these errors and any consequent inconvenience to authors and readers.

## Supplementary Material

