# Supplementary material for: Helium nanodroplets as an efficient tool to investigate hydrogen attachment to alkali cations
Source: Phys Chem Chem Phys. 2022 Nov 17;25(1):462–70. doi: 10.1039/d2cp03841b (PMC9768848; doi:10.1039/d2cp03841b)
Supplement: CP-025-D2CP03841B-s001 [file CP-025-D2CP03841B-s001.pdf]

Electronic Supplementary Material (ESI) for Physical Chemistry Chemical Physics.
This journal is © the Owner Societies 2022

Supplementary Information: "Helium nanodroplets as an efficient tool to investigate hydrogen attachment to alkali cations"

Siegfried Kollotzek ,[*,†] José Campos-Martínez,[*,‡] Massimiliano Bartolomei,[‡] Fernando Pirani,[¶] Lukas Tiefenthaler ,[†] Marta I. Hernández,[‡] Teresa Lázaro,[‡] Eva Zunzunegui-Bru ,[‡] Tomás González-Lezana,[‡] Javier Hernández-Rojas,[§] José Bretón,[§] Olof Echt,[‖] and Paul Scheier[†]

†*Universität Innsbruck, Institut für Ionenphyisk und Angewandte Physik, Technikerstraße 25, 6020 Innsbruck, Austria*

‡*Instituto de Física Fundamental, Consejo Superior de Investigaciones Científicas (IFF-CSIC), Serrano 123, 28006 Madrid, Spain*

¶*Dipartimento di Chimica, Biologia e Biotecnologie, Università di Perugia, Perugia, Italy*

§*Departamento de Física and IUdEA, Universidad de La Laguna, 38205, La Laguna, Tenerife, Spain*

‖*Department of Physics, University of New Hampshire, Durham, NH 03824, USA and Universität Innsbruck, Institut für Ionenphyisk und Angewandte Physik, Technikerstraße 25, 6020 Innsbruck, Austria*

E-mail: Siegfried.Kollotzek@uibk.ac.at; jcm@iff.csic.es

Supplementary Information

Experimental Pressure Scans and Mass Spectra

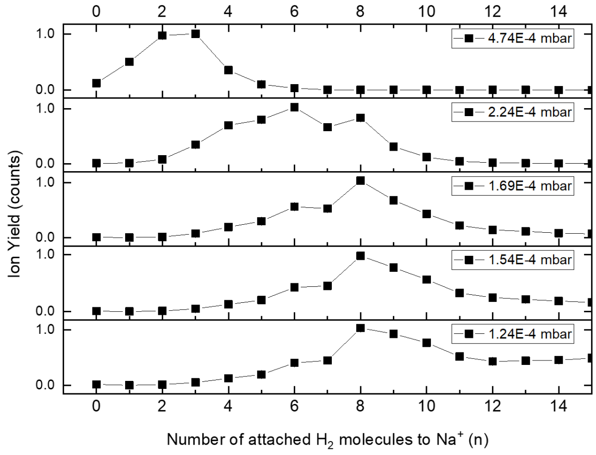

Figure S1: Evolution of the hydrogen decoration of the sodium monomer for five different pressures. It can be seen that "magic numbers" are pressure independent. Especially 6 and 8 hydrogen molecules appear strongly unaffected.

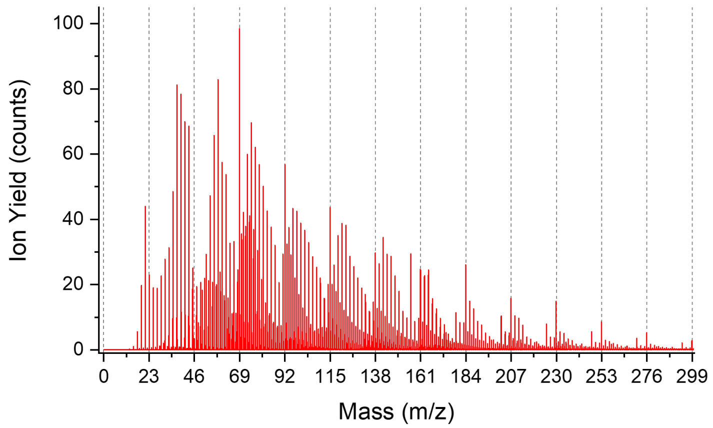

Figure S2: Example of mass spectrum of H_2 decorated positively charged sodium. Fig.(2) in the main text, is a zoom of this spectrum up to ≈ mass 50 (m/z)

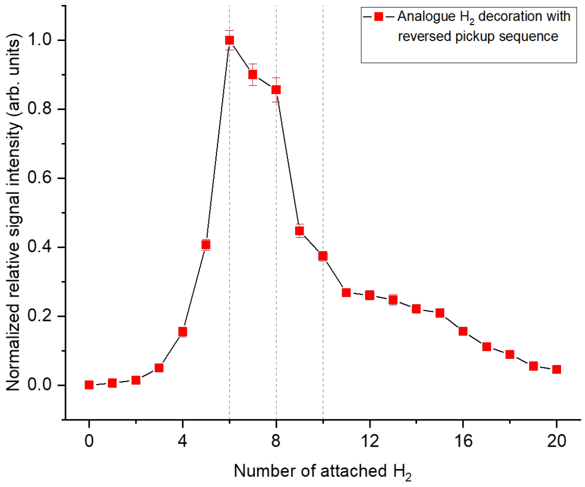

Figure S3: Experimental results for the reversed (H_2 doping before sodium pick up with subsequent collision with helium in the evaporation chamber), the graph shows the presence of magic number (mainly 6,8,10) similar to the results discussed in the main manuscript for the regular pick up sequence.

Potential Energy Surfaces

The $Na^+(H_2)_n$ Case

The total interaction potential for $Na^+(H_2)_n$ is assumed to be a sum of two-body (2B) and three-body (3B) interactions:

$$V\left[\mathrm{Na}^+(\mathrm{H}_2)_n\right] = \sum_{i=1}^{n} V^{2B}\left[\mathrm{H}_2^{(i)} - \mathrm{Na}^+\right] + \sum_{i<j}^{n} V^{2B}\left[\mathrm{H}_2^{(i)}{-}\mathrm{H}_2^{(j)}\right] + \sum_{i<j}^{n} V^{3B}\left[\mathrm{H}_2^{(i)}{-}\mathrm{Na}^+{-}\mathrm{H}_2^{(j)}\right]. \tag{1}$$

For the $[H_2^{(i)} - Na^+]$ (2B) term, and considering each H_2 molecule as a rigid rotor (RigRot), the potential is represented analytically by a non-covalent contribution (V_{NC}), described using the atom-bond model[1] and an Improved Lennard Jones (ILJ) formulation, plus an electrostatic (V_{elec}) contribution. More details on the determination and expression of both contributions are given in Ref.[2]

As for the $[\mathrm{H}_2^{(i)}{-}\mathrm{H}_2^{(j)}]$ (2B) term, it is also expressed as a sum of non-covalent plus electrostatic contributions (following the procedure indicated in Ref.[3]) being each one, within the RigRot approximation, depending only on Jacobi coordinates: the modulus of the vector $\vec{r}_{ij}$ between the center of mass of the two diatomic molecules, and the angles θ_a, θ_b between

the previous vector and H_2 internuclear distances, plus ϕ_{ab}, the torsion angle.

Table S1: Optimized parameters for the $(H_2)_n$-Na^+ PES, within the rigid rotor and the pseudo-atom approximations (see Ref.([3]) for a more complete account on parameters and procedures for the pseudo-atom approach). Distances $r_e^{\perp}$, $r_e^{\parallel}$, $\bar{r}_e$, and the internuclear $H-H$ distance ρ are in Å, while well depths $\varepsilon^{\perp}$, $\varepsilon^{\parallel}$ and $\bar{\varepsilon}$ are in meV. Common for the two approximations are m and β (dimensionless) and α, the H_2 average polarizability (from Ref.[4]times 1.05), in Å^3. H_2 partial charges are in units of the proton charge; q_H is located on top of the H nuclei and q_{CM} at the bond center.

			Rigid rotor				Pseudo-atom	
Dimer	m	β	$r_e^{\perp}$	$r_e^{\parallel}$	$\varepsilon^{\perp}$	$\varepsilon^{\parallel}$	$\bar{r}_e$	$\bar{\varepsilon}$
H_2-Na^+	4	4.7	2.51	2.61	95.93	126.87	2.54	106.24
H_2-H_2	6	7.0	3.46	3.50	1.30	2.00	3.47	3.07
α	0.8263							
ρ			0.76664					
H_2 charges			q_H =0.45955,		$q_{CM}=-2q_H$			

Finally, the third term in Eq.1 corresponds to the interaction between the dipoles that the cation induces in the hydrogen molecules i and j as employed in previous studies[4–6]

$$V^{3B}[H_2^{(i)}-Na^+-H_2^{(j)}] = -\frac{\alpha^2}{4}[3r_i g_3(r_j)g_5(r_{ij}) + 3r_j g_3(r_i)g_5(r_{ij}) -g_3(r_i)g_3(r_j)g_1(r_{ij}) - 6g_1(r_i)g_1(r_j)g_5(r_{ij}) -2g_1(r_i)g_3(r_j)g_3(r_{ij}) - 2g_3(r_i)g_1(r_j)g_3(r_{ij})] \tag{2}$$

where $g_n(r) = r^{-n}$, r_i and r_j are the distances between (the center of mass of) $H_2(i)$ and $H_2(j)$ to Na^+, respectively, r_{ij} is the distance between the hydrogen molecules, and α is the polarizability of H_2. This magnitude is in fact a tensor which in the molecular frame is diagonal with two distinct components $\alpha_{\parallel}$ and $\alpha_{\perp}$, so in principle the polarizability varies with the orientation of the molecule with respect to the cation. We have found that this anisotropic contribution is negligible (less than 0.1 meV difference in the total energy as compared with using a spherically averaged polarizability, for $(H_2)_2Na^+$ at equilibrium). Hence

we have adopted, for the averaged polarizability, the reference value[4] $\alpha = 0.7870$ Å^3, that has been multiplied by 1.05 to get a better comparison with ab-initio estimations. As in previous cases[3] our potential model provides a good agreement with *ab initio* (supermolecular) estimations obtained at the CCSD(T)/CBS level for this system.

Therefore, the 3B term can also be used in a common approximation within this context which is the treatment of the diatomic H_2 molecule as a pseudo atom (Psat). Within this approach the parameters needed in the ILJ atom-atom interaction fit, are given by $\bar{r}_e = (2\, r_e^{\perp} + r_e^{\|})/3$ and $\bar{\epsilon}_e = (2\, \epsilon_e^{\perp} + \epsilon_e^{\|})/3$.

Besides, within this Psat model, in a consistent way the $[\mathrm{H}_2^{(i)}\text{–}\mathrm{H}_2^{(j)}]$ interaction is also taken as an average over all dimers' orientations and represented by atom-atom ILJ functions, as that of Eq.(2) of *Supplementary Information* of Ref.,[3] where a more detailed information can be found on the methodology and also the value of potential parameters which are the same than the one employed here, for well depths and equilibrium positions.

A compilation of the optimized values of all the parameters involved in the analytical RigRot and Psat PESs are given in Table S1.

The $Na_2^+(H_2)_n$ Case

In this case only (2B) contributions are considered and the potential interaction is expressed as follows:

$$V\left[\mathrm{Na}_2^+(\mathrm{H}_2)_n\right] = \sum_{i=1}^{n} V^{2B}\left[\mathrm{H}_2^{(i)} - \mathrm{Na}_2^+\right] + \sum_{i<j}^{n} V^{2B}\left[\mathrm{H}_2^{(i)}\text{–}\mathrm{H}_2^{(j)}\right] \quad (3)$$

The second term in Eq.(3), is the the same as in the case of the Na^+ monomer, while the interaction between the Na_2^+ and the H_2 molecules is given, as usually, as a sum of van der Waals non-covalent (NC) plus electrostatic interactions, i.e. $V^{2B}\left[\mathrm{H}_2^{(i)} - \mathrm{Na}_2^+\right] = V_{NC} + V_{elec}$.

Both interactions depend on the distance R between the centers of mass of the two diatomic under consideration, the angles Θ_a, Θ_b between the (vector) $\boldsymbol{R}$ and the vectors defining the bond lengh of Na_2^+ and H_2 respectively, and finally Φ, the dihedral angle, the common Jacobi coordinates for two diatomic molecules. Please note that we also use (θ, Φ), with other meaning for the cluster structure, but we think they are easily differentiate because they are used in very different context.

The V_{NC} term is evaluated as a sum of bond-bond pairwise interactions, which better represent the Na_2^+ bond polarizability and its contribution to the whole interaction.[7,8]

Specifically, these interactions are represented by the Improved Lennard-Jones potential expression[8,9]

$$V_{vdW}(R,\gamma) = \varepsilon(\gamma)\left[\frac{m(\gamma)}{n(R,\gamma)-m(\gamma)}\left(\frac{R_m(\gamma)}{R}\right)^{n(R,\gamma)} - \frac{n(R,\gamma)}{n(R,\gamma)-m(\gamma)}\left(\frac{R_m(\gamma)}{R}\right)^{m(\gamma)}\right] \quad (4)$$

and γ denotes collectively the triplet of angles Θ_a, Θ_b, Φ while $\epsilon(\gamma)$ and $R_m(\gamma)$ are the well depth and the equilibrium distance, while

$$n(R,\gamma) = \beta(\gamma) + 4.0\left(\frac{R}{R_m(\gamma)}\right)^2 \quad (5)$$

where $\beta(\gamma)$ represents and an additional parameter (with respect to the conventional Lennard-Jones one), that provides more flexibility in the description of the repulsive wall of the interaction.

Table S2: Optimized parameters for the $Na_2^+(H_2)_n$ bond-bond non-covalent interaction.

h

Configurations	ϵ (meV)	R_m (Å)	β	m
H	5.75	4.37	6.10	4.0
X	5.75	4.37	6.10	4.0
T_b	34.44	4.73	8.50	6.0
T_a	7.41	4.37	6.10	4.0
L	44.74	4.61	6.10	6.0

The angular dependence of V_{vdW} is obtained by representing the ε, R_m, β and m potential parameters in a spherical harmonic expansion[7,10,11]

$$\varepsilon(\gamma) = \varepsilon^{000} + \varepsilon^{202}A^{202}(\gamma) + \varepsilon^{022}A^{022}(\gamma) + \varepsilon^{220}A^{220}(\gamma) + \varepsilon^{222}A^{222}(\gamma) \quad (6)$$

$$R_m(\gamma) = R_m^{000} + R_m^{202}A^{202}(\gamma) + R_m^{022}A^{022}(\gamma) + R_m^{220}A^{220}(\gamma) + R_m^{222}A^{222}(\gamma) \quad (7)$$

$$\beta(\gamma) = \beta^{000} + \beta^{202}A^{202}(\gamma) + \beta^{022}A^{022}(\gamma) + \beta^{220}A^{220}(\gamma) + \beta^{222}A^{222}(\gamma) \quad (8)$$

$$m(\gamma) = m^{000} + m^{202}A^{202}(\gamma) + m^{022}A^{022}(\gamma) + m^{220}A^{220}(\gamma) + m^{222}A^{222}(\gamma) \quad (9)$$

where the coefficients of the bipolar spherical harmonics $A^{L_1L_2L_3}(\gamma)$ can be obtained as illustrated in Ref.[7] from the ε, R_m, β and m values corresponding to specific angular configurations of the H_2-Na_2^+ dimer.

These values are reported in Table S2 for the (H, X, T_a, T_b and L) dimer configurations, which correspond to the angular triplets $\gamma = (\Theta_a, \Theta_b, \Phi)$=$H(90^o, 90^o, 0^o)$, $X(90^o, 90^o, 90^o)$, $T_a(90^o, 0^o, 0^o)$, $T_b(0^o, 90^o, 0^o)$, $L(0^o, 0^o, 0^o)$, respectively. A method to estimate zeroth order values of the ϵ and R_m reported in Table S2 from diatomic (or molecular bond) polarizability values is illustrated in Appendix A of ref[7].

All the parameters in Table S2 have been optimized on the basis of the comparison of analytical interaction energies with the ab initio estimations reported in Fig.S4.

Regarding the V_{elec} electrostatic contribution, it has been obtained from coulombic interactions involving point charges conveniently distributed on both monomers. For the H_2 monomer the charges are those reported in TableS1. As for Na_2^+, the corresponding charges are $q_{Na} = 0.93927\, a.u.$ and $q_{cm} = -0.87854\, a.u.$, for the Na atoms and center of mass, respectively. They have been obtained at a ACPF/AVTZ level, following the procedure given in Ref.[12] and corresponding to a diatomic equilibrium distance (optimized at the CCSD(T)/AVTZ level) of 3.7113 Å: these charges are those compatible with a calculated quadrupole moment of 23.1 a.u. plus a positive charge (+1) placed in correspondance of the molecule center of mass to account for the global charge of the cation.

A comparison between the interaction profiles for the (H, X, T_a, T_b and L) dimer configurations, obtained by using the above formulation and from ab initio estimations carried out at the RCCSD(T)/CBS level of theory (and following the guidelines as in Ref.[2]) can be seen in Fig.S4: it can be appreciated that the analytical representation is capable to reproduce very well the main interaction features of the $Na_2^+ - H_2$ dimer.

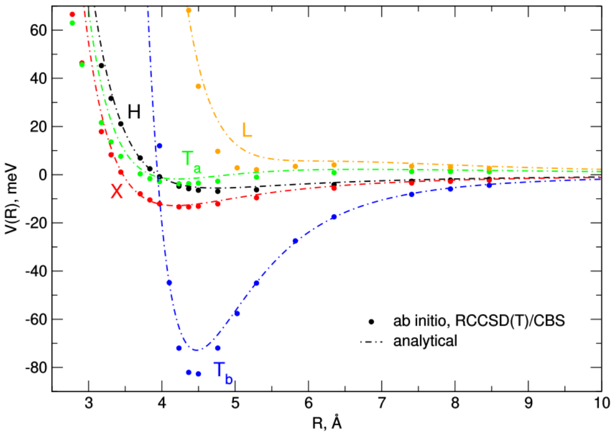

Figure S4: Comparison of ab initio interaction energies and their analytical representation for selected configurations (see text) of the $Na_2^+H_2$ dimer. R is the distance between the centers of mass. Notice that T_b denotes the configuration with Na_2^+ aligned along R

Classical Structures and Local Minima

In order to study the global minima we have used two different algorithms, a Basin Hoping (BH) technique[13,14] using the pseudo atom model and a classical Monte Carlo one in the case of the rigid rotor model for the H_2 molecule.

Favorable structures for $Na^+(H_2)_n$ clusters up to $n = 14$ were found by unbiased BH global optimization technique.[13] This stochastic algorithm explores the potential energy surface and locates the global minimun using random moves on local minima, following a Metropolis algorithm. Suitable parameters for the BH are optimization temperature ($k_BT = 3\,meV$), where k_B is the Boltzmann constant, and number of steps (2×10^4), The same global minimum was located in all trajectories.

Within the rigid rotor model for the H_2 molecules which surround Na^+ or Na_2^+ dopants, we have run classical simulated annealing Monte Carlo calculations[15] which involve both translational and rotational moves and equilibration at ever-lower temperatures (typically starting from 8 K and cooling to about 0.5 K). The structures so obtained correspond to the putative global minima of the interaction and are then used as suitable initial configurations for the Diffusion Monte Carlo calculations.

Diffusion Monte Carlo

We have used the code developed by Sandler and Buch[16,17] which has been succesful in the study of various molecular clusters,[3,18,19] and specially suitable for the treatment of rigid molecules. For a given cluster size, eight runs were usually performed, each of them involving eigth generations of a descendant weighting procedure,[20] and averages of the final energies of the different runs are computed together with associated standard deviations. Between 20000 and 25000 replicas were propagated with time steps ranging from 100.0-12.5 a.u., for in between 2500-20000 steps, up to reach convergence. The initial population was typically built from a Gaussian distribution centered in the minimum of the PES, previously determined from the classical structures.

Cluster energies were easily converged for the smaller clusters ($N < 14$), with standard deviations of about 0.03-1.0 meV.

Path Integral Monte Carlo

The PIMC method is the same as in previous applications for the study of similar ion doped helium clusters[19,21] and it has been described elsewhere before.[22] The energy of each cluster is estimated by means of the thermodynamic approach developed by Barker:[23]

$$\langle E \rangle_{\text{thermo}} = \frac{3N}{2\tau} - \left\langle \sum_{\alpha=0}^{M-1} \sum_{i=1}^{N} \frac{(\mathbf{r}_i^{\alpha} - \mathbf{r}_i^{\alpha+1})^2}{4M\lambda_m \tau^2} - V \right\rangle, \quad (10)$$

where $\lambda_m = \hbar^2/2m$, m is the mass of H_2 or Na^+ and $\tau = \beta/M$, with $\beta = (k_B T)^{-1}$. The expression in Eq. (10) consists on a first term describing the classical kinetic energy multiplied by M (the number of quantum beads in the factorization of the density matrix[19,21]) and a second term with the average of the energy due to the spring-like interaction assumed between consecutive beads in the same ring describing a specific particle and of the potential energy V. The PIMC calculation has been performed at 2 K and $M = 200$ have been enough to ensure converged results, which are moved in groups of 10 following a staging method.[24,25]

Typical Structures and Relevant Angles

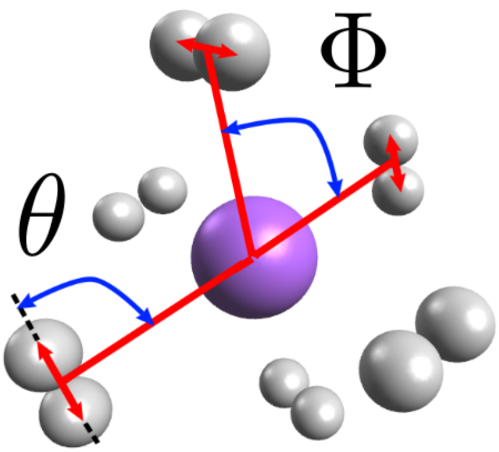

Figure S5: Classical structure corresponding to $Na^+(H_2)_6$. Relevant angles: θ, formed by the vector bondlength and the vector joining the center of mass of a monomer H_2 with the cation Na^+ and Φ, the angle formed by vectors joining the sodium cation and the center of mass of any two monomers.

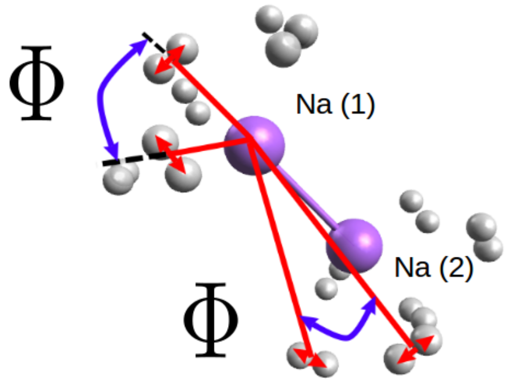

Figure S6: Classical structure corresponding to $Na_2^+(H_2)_{12}$. Here only the Φ angles, between vectors directed from one of the sodium atoms (label $Na(1)$) to any two momoners H_2 are displayed. Note that there are two different groups of hydrogen monomers one of them around one of the sodium, $Na(1)$, and another group around the other sodium $Na(2)$, both of them forms the sodium dimer N_2^+.

References

(1) Pirani, F.; Albertí, M.; Castro, A.; Teixidor, M. M.; Cappelletti, D. Atom-Bond Pairwise Additive Representation for Intermolecular Potential Energy Surfaces. *Chem. Phys. Lett.* **2004**, *394*, 37–44.

(2) Bartolomei, M.; González-Lezana, T.; Campos-Martínez, J.; Hernández, M. I.; Pirani, F. Complexes of Alkali Metal Cations and Molecular Hydrogen: Potential Energy Surfaces and Bound States. *J. Phys. Chem. A* **2019**, *123*, 8397–8405.

(3) Ortiz de Zárate, J.; Bartolomei, M.; González-Lezana, T.; Campos-Martínez, J.; Hernández, M. I.; Pérez de Tudela, R.; Hernández-Rojas, J.; Bretón, J.; Pirani, F.; Kranabetter, L. et al. Snowball formation for Cs^+ solvation in molecular hydrogen and deuterium. *Phys. Chem. Chem. Phys.* **2019**, *21*, 15662–15668.

(4) Olney, T. N.; Cann, N. M.; Cooper, G.; Brion, C. E. Absolute Scale Determination for Photoabsorption Spectra and the Calculation of Molecular Properties Using Dipole Sum Rules. *Chem. Phys.* **1997**, *223*, 59–98.

(5) Liu, M. M.; Wu, M. S.; Han, H. L.; Shi, T. Y. Hyperspherical Coupled Channel Calculations of Energy and Structure of ^4He-^4He-Li^+ and its isotopic combination. *Journal of Chemical Physics* **2016**, *145*, 034304.

(6) Rastogi, M.; Leidlmair, C.; An der Lan, L.; Ortiz de Zárate, J.; Pérez de Tudela, R.; Bartolomei, M.; Hernández, M. I.; Campos-Martínez, J.; González-Lezana, T.; Hernández-Rojas, J. et al. Lithium ions solvated in helium. *Phys. Chem. Chem. Phys.* **2018**, *20*, 25569–25576.

(7) Cappelletti, D.; Pirani, F.; Bussery-Honvault, B.; Gómez, L.; Bartolomei, M. A Bond-Bond Description of the Intermolecular Interaction Energy: the Case of Weakly Bound N_2-H_2 and N_2-N_2 Complexes. *Phys. Chem. Chem. Phys* **2008**, *10*, 4281.

(8) Hong, Q.; Sun, Q.; Pirani, F.; Valentín-Rodríguez, M. A.; Hernández-Lamoneda, R.; Coletti, C.; Hernández, M. I.; Bartolomei, M. Energy exchange rate coefficients from vibrational inelastic $O_2(^3\Sigma_g^-) + O_2(^3\Sigma_g^-)$ collisions on a new spin-averaged potential energy surface. *The Journal of Chemical Physics* **2021**, *154*, 064304.

(9) Pirani, F.; Brizi, S.; Roncaratti, L.; Casavecchia, P.; Cappelletti, D.; Vecchiocattivi, F. Beyond the Lennard-Jones Model: A Simple and Accurate Potential Function Probed by High Resolution Scattering Data Useful for Molecular Dynamics Simulations. *Phys. Chem. Chem. Phys* **2008**, *10*, 5489–5503.

(10) Bartolomei, M.; Pirani, F.; LaganÃ , A.; Lombardi, A. A full dimensional grid empowered simulation of the CO2 + CO2 processes. *Journal of Computational Chemistry* **2012**, *33*, 1806–1819.

(11) Karimi-Jafari, M. H.; Ashouri, M.; Yeganeh-Jabri, A. Coping with the anisotropy in the analytical representation of an ab initio potential energy surface for the Cl2 dimer. *Phys. Chem. Chem. Phys.* **2009**, *11*, 5561–5568.

(12) Bartolomei, M.; Carmona-Novillo, E.; Hernández, M. I.; Campos-Martínez, J.; Hernández-Lamoneda, R. Long Range Interaction for Dimers of Atmospheric Interest: Dispersion, Induction and Electrostatic Contributions for O_2-O_2, N_2-N_2 and O_2-N_2. *J. Comp. Chem.* **2011**, *32*, 279–290.

(13) Wales, D. J.; Doye, J. P. K. Global optimization by basin-hopping and the lowest energy structures of Lennard-Jones clusters containing up to 110 atoms. *J. Phys. Chem. A* **1997**, *101*, 5111–5116.

(14) Hernández-Rojas, J.; Wales, D. J. Global minima for rare gas clusters containing one alkali metal ion. *J. Chem. Phys.* **2003**, *119*, 7800–7804.

(15) Wille, L. Minimum-energy configurations of atomic clusters: new results obtained by simulated annealing. *Chemical Physics Letters* **1987**, *133*, 405–410.

(16) Buch, V. Treatment of Rigid Bodies by Diffusion Monte-Carlo. Application to the Para-H_2...H_2O and Ortho-H_2...H_2O Clusters. *J. Chem. Phys.* **1992**, *97*, 726–729.

(17) Sandler, P.; Buch, V. *General purpose QCLUSTER program for Rigid Body Diffusion Monte Carlo simulation of an arbitrary molecular cluster*; (private communication), 1999.

(18) Kolmann, S. J.; D'Arcy, J. H.; Jordan, M. J. T. Quantum Effects and Anharmonicity in the H_2-Li^+-benzene complex: A Model for Hydrogen Storage Materials. *J. Chem. Phys.* **2013**, *139*, 234305.

(19) Rastogi, M.; Leidlmair, C.; An der Lan, L.; Ortiz de Zárate, J.; Pérez de Tudela, R.; Bartolomei, M.; Hernández, M. I.; Campos-Martínez, J.; González-Lezana, T.; Hernández-Rojas, J. et al. Lithium ions solvated in helium. *Phys. Chem. Chem. Phys.* **2018**, *20*, 25569–25576.

(20) Suhm, M. A.; Watts, R. O. Quantum Monte Carlo Studies of Vibrational States in Molecules and Clusters. *Phys. Rep.* **1991**, *204*, 293 – 329.

(21) Pérez de Tudela, R.; Martini, P.; Goulart, M.; Scheier, P.; Pirani, F.; Hernández-Rojas, J.; Bretón, J.; Ortiz de Zárate, J.; Bartolomei, M.; González-Lezana, T. et al. A combined experimental and theoretical investigation of Cs^+ ions solvated in He_N clusters. *J. Chem. Phys.* **2019**, *150*, 154304.

(22) Rodríguez-Cantano, R.; González-Lezana, T.; Villarreal, P. Path integral Monte Carlo investigations on doped helium clusters. *Int. Rev. Phys. Chem.* **2016**, *35*, 37–68.

(23) Barker, J. A. A quantum-statistical Monte Carlo method; path integrals with boundary conditions. *J. Chem. Phys.* **1979**, *70*, 2914.

(24) Ceperley, D. M. Path integrals in the theory of condensed helium. *Rev. Mod. Phys.* **1995**, *67*, 279.

(25) Pollock, E. L.; Ceperley, D. M. Simulation of quantum many-body systems by path-integral methods. *Phys. Rev. B* **1984**, *30*, 2555–2568.
